# Improved Design via Simulation of Micro-Modified PVDF and Its Copolymer Energy Harvester with High Electrical Outputs

**DOI:** 10.3390/s20205834

**Published:** 2020-10-15

**Authors:** Yizhi Liu, Ziyu Huang, Chen Liu

**Affiliations:** Department of Astronautic Science and Mechanics, Harbin Institute of Technology, Harbin 150001, China; 1161800308@stu.hit.edu.cn (Z.H.); 19S018019@stu.hit.edu.cn (C.L.)

**Keywords:** P(VDF-TrFE), PVDF, piezoelectric nanogenerator, micro-modified, periodical changing load

## Abstract

In this work, micro-modified polyvinylidene fluoride (PVDF) and its copolymer poly(vinylidene-trifluoroethylene) (P(VDF-TrFE)) with salient enhancement in current output are demonstrated. The influence of surface-modified structure characteristics on electrical properties of energy harvester is systematically analyzed based on the finite element method. For vertical load mode, eight structures consisting of banded and disjunctive groups are compared to evaluate the voltage performance. The cylinder is proved to be the best structure of 3.25 V, compared to the pristine structure of 0.99 V (P(VDF-TrFE)). The relevant experiment has been done to verify the simulation. The relationship between radius, height, force and distance to the voltage output of the cylinder allocation is discussed. For periodical changing load mode, the cylinder modified structure shows a conspicuous enhancement in current output. The suitable resistance, current–voltage and frequency, the relationship between loading speed and current, and the ductility of current loading are studied. For 30 kHz, the peak current is 20 times larger than the flat plate structure. Tip shape mode and fusiform shape mode are found, which show the different shapes of the peak current-frequency curves. Four electrical loading circuit properties are also discussed: the suitable resistance of the system, synchronism of current and voltage, time delay nature of energy harvester and current-loading relationship. The simulation results can provide some theoretical basis for designing the energy harvester and piezoelectric nanogenerator (PENG).

## 1. Introduction

The declining future of fossil fuel has brought about much interest in sustainable new energy resources. The urgency of state-of-the-art small scale electronic devices has promoted the enthusiasm for portable, light-weighted, ductile, and multifunctional energy generators. The small-scale feature makes it difficult for such devices to charge the inside battery, therefore self-powering material, which can utilize mechanical energy, such as piezoelectric material, has been given much attention. Taking advantage of the mechanical energy to support small scale devices is possible [1,2,3,4,5]. A great amount of mechanical energy in nature has been wasted, such as airflow, human mechanical motion, and tire scrolling [6,7,8]. The type of material and the shape of the piezoelectric energy nanogenerator (PENG) are the fundamental considerations here. Polyvinylidene fluoride (PVDF) and its copolymer poly(vinylidene-trifluoroethylene) (P(VDF-TrFE)) are researched in this paper because of their excellent properties. (1) High piezoelectric is constant. The piezoelectric constants are almost ten times higher than quartz [8,9,10,11,12]. (2) Perfect flexibility. These can be fabricated into 5 μm to 1 mm thin film with a large area [13,14,15,16]. (3) Low acoustic impedance. Their acoustic impedance is similar to the human body’s impedance, which is only 1/10 of PZT (lead zirconate titanate) [17,18,19,20]. (4) Perfect thermal stability. The fluorine atoms inside enhance its chemical stability and fatigue resistance [21,22,23,24,25,26]. (5) High dielectric strength [27,28,29] and low mass density [30,31,32]. They can withstand very high electric field strength, on the contrary, the ceramic piezoelectric materials will depolarize easily at high electric field strength.

Based on the piezoelectric material, PENG has developed rapidly in recent years. Wang et al. combine PDMS/MWCNT thin composite membrane with P(VDF-TrFE) nanofibers, which realizes both piezoelectric and triboelectric energy harvesting [33]. Rashmi et al. [34] developed a new Si bulk-based micromachining process for MEMS fabrication. The simulation agrees well with the experiment outcome, and the method has the potential for low-frequency energy harvester development. You et al. [35] has fabricated aligned P(VDF-TrFE) nanofibers using the electrospinning technique. The SEM images show that the nanofibers are highly ordered. Rashmi et al. have proposed a very low frequency PENG design using P(VDF-TrFE). Three design types are tested, and the frequency of 10 μm × 5 μm × 3 μm type is 42.68 Hz. The research shows that P(VDF-TrFE) is useful for very low frequency PENG design [36].

As mentioned before, the shape is essential in PENG design. Based on the simulation method, much research has been done. Qin et al. have designed a sandwich structure PENG with Mo and Pt as electrode and ZnO as a piezoelectric layer [37]. This structure shows a voltage outcome of around 95 mV, a current density of 35 μAcm^−2^, and an energy density of 5.1 mW/cm^2^. Chang et al. have designed a perovskite-crystal-structured PENG using barium titanate (BaTiO_3_) nanotube by a controlled focused ion beam-assisted method [38]. This structure is flexible and produces voltage and current output of 1 V and 20 nA. Zurkinden et al. have designed a foam core structure of PVDF with a length of 30 mm, which is practicable in the seabed with wave frequency of 0.89 Hz. The interaction between the structure and fluid and the mechanical input and the electrical output are all examined by COMSOL. The power output is faint, but it provides a valuable model for future wave energy harvesting [39]. Choi et al. have designed a structure of PVDF-coated ZnO nanowire array. The mechanical properties are shown by atomic force microscopy (AFM) and finite element method (FEM) simulation, and the electronic properties are researched by electrostatic force microscopy and direct I-V measurements. The shape modification largely enhances the voltage output from 0.15 V to 0.4 V comparing to pristine PVDF, the ZnO pillar inside could enhance its mechanical properties [40]. Han et al. [41] have provided an R-shaped PDMS (polydimethylsiloxane)-PVDF, which facilitates the combination of piezoelectric and triboelectric properties. Simply pressing and releasing could achieve a power density of 10.95 (piezoelectric) and 2.04 (triboelectric) mW/cm^3^. Jung et al. have created wearable PENG with a curved shape of a pair of arc brackets, which could produce a peak voltage of 120 V and a current of 700 mA. It works well at low-frequency. At 50 Hz, it produces a peak voltage of 55 V [42]. Pobering et al. have produced a PENG which works in a flowing media such as air or water. The maximum power of the structure could reach 0.1 mW. The structure is improved by adjusting the length of the cantilever and the scale of the connection resistance. The voltage could increase with the flowing velocity [43]. Chen et al. have proven that cylinder micro-pillar shape is better than pristine PENG, with an increase voltage output from 1.85 V to 13.1 V [44]. These structures are manufacturable through 3D printing or molding.

As far as the authors know, this is the first systematic research about the influence of various structures on electric output, and this is the first simulation on periodical changing load on PENG. In this work, the vertical load mode and periodical changing load mode on micro-modified PENG are researched, respectively. Under vertical load mode, eight structure designs are compared for evaluation of the voltage output. Relevant experiment is done to verify the simulation. The distance between the cylinder, radius, force, and the height of the cylinder are discussed. Under periodical changing load mode, the period peak value of the current response on the cylinder modified-structure is found and compared to the flat plate structure. Different modes of current responses are discussed. The suitable resistance, synchronism of current and voltage, time delay nature and current-loading relationship of PENG are discussed. All of the above studies are based on simulation using COMSOL 5.4, and most of them are compared to the existing research. The structure mentioned in this paper is manufacturable through 3D printing or molding.

**Figure 1:**
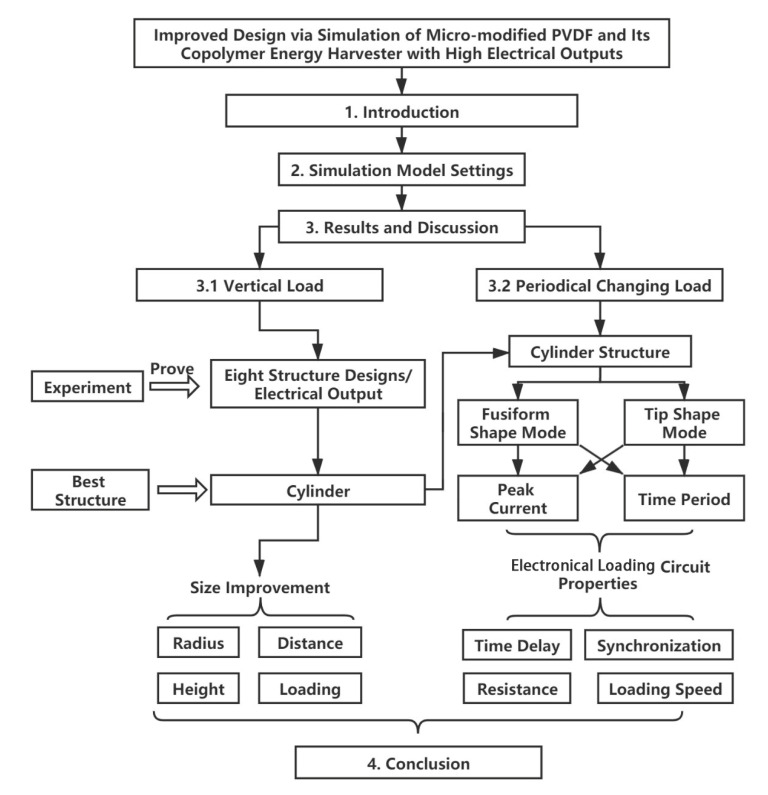
Main structure of the paper.

The main structure of the paper is shown in Figure 1. The first section introduces the main idea of the paper, the second section describes the simulation model settings, and the third section contains the results and the discussion, consisting of the vertical load mode and the periodical changing load.

## 2. Simulation Model Settings

### 2.1. Material Properties

Part of the material matrix of PVDF can be found in the COMSOL 5.4 built-in parameter library. The key parameters, such as d_33_ (piezoelectric constant), are checked carefully through paper research. The ε_33_ of PVDF and P(VDF-TrFE) are 7.6 and 8 [45], respectively. The d_33_, one of the most important constants of the piezoelectric material, is 15 pC/N and 17 is for pC/N for PVDF and P(VDF-TrFE), respectively.

### 2.2. Constitutive Equations

(1) $$T=c^{E}S-eE$$

(2) $$D=eS+\varepsilon^{S}E$$

where *T* is stress, *c^E^* is the elastic matrix in constant strain mode, *S* is the strain, *e* is piezoelectric constant, *E* is electric field intensity, *D* is electric displacement, $\varepsilon^{S}$ is dielectric constant in the constant electric field.

### 2.3. Mesh and Boundary Condition

**Figure 2:**
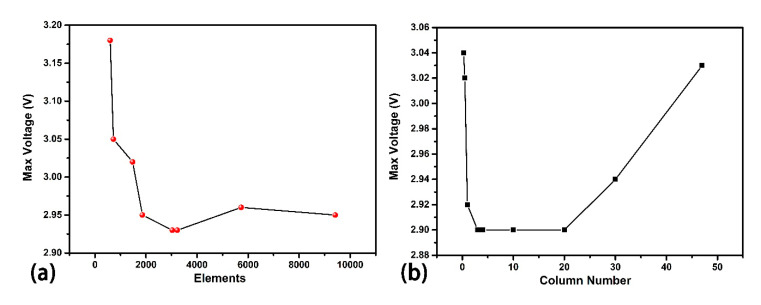
(**a**) Mesh Converge Diagram of the cylinder modified structure, of which bottom shape is 200 μm × 200 μm and the cylinder diameter is 30 μm and the height is 30 μm. The x axis is the element number after meshing, and the y axis is the max voltage of each mesh type. (**b**) Column Number Independence test. The cylinder modified structure is tested under different column number. For example, column number 4 means 4 × 4 columns locate on corresponding support base. Column number 0.5 means a semi-cylinder locates on the support base using periodic condition.

The free tetrahedral mesh is applied. The max unit scale is 80 μm, and the minimum scale is 14 μm, the maximum unit growth rate is 1.5, and the curvature factor is 0.6. The total structure (for cylinder modified structure in vertical load mode) consists of 9414 elements. The bottom of the vertical load mode is fixed, and the 10 μm × 200 μm side of the cantilever of periodical changing load mode is fixed. Figure 2a shows the mesh converge message of the cylinder modified structure under vertical load mode. As the mesh becomes finer, the element number increases, and the max voltage value converges. The element number used in this research is 9414, which sufficiently reaches the convergence. The mesh method in COMSOL is “physics-controlled mesh” and the element size degree is “normal”.

### 2.4. Vertical Load Model

#### 2.4.1. Device Structure

The large quantity of the structure design is one distinctive feature of this paper. Eight designs that are popular in shape design are brought up and calculated using COMSOL 5.4.

The diameter of all circles in these structures is 30 μm, and the side length of all of the square is 30 μm, and the height of the rectangular column, rectangular groove, and the cylinder is 30 μm. The radius of the half-cylinder is 15 μm, considering the limitation of the total structure, and the triangular prism’s triangular is an equilateral triangle with a height of 30 μm. The width of the rectangular groove is 30 μm, the height is 30 μm, and the distance between two geometric centers is 45 μm. The distances (between two geometric centers) of half-cylinder and triangular prism are all 45 μm.

Generally, the structure is divided into two parts, the banded structure, and the disjunctive structure. The disjunctive part denotes a semi-sphere, pyramid, rectangular column, and cylinder. The banded part denotes half-cylinder, triangular prism, and rectangular groove. All of the shapes consist of two parts, the support base, and the function top shape. The variable control is the key consideration in vertical load mode design. The thickness of all of the bases is set as 10 μm. The length and the width of the base are all set as 200 μm × 200 μm, which locates four rows and four columns or just four rows. A Column Number Independence test is done using cylinder modified structure. Different column number is tested. As shown in Figure 2b, the result shows the fluctuation due to column number is small enough (0.1/3 ≈ 3%). Considering the aesthetics of the figure and the simplicity of computing, a 4 × 4 column is chosen for further research. The function structures are located in the middle part of the base structures to avoid aberrant voltage output derived from edge collapse. The five-circle tangent principle is applied to specific structures. This principle means the circle center locates in four vertices of a square, with the same circle inscribed in the middle. Every four outer circle represents a real cylinder, and the inscribed circle is just an interval. It is also valuable to note that these eight structures are manufacturable using 3D printing or molding.

#### 2.4.2. Loading Settings

The loading force on the function top is all set as 5 mN. For the pristine, cylinder, rectangular column, and rectangular groove structure, the force is loaded on each function top. For hemisphere, half cylinder, triangular prism, and pyramid structure, the force is loaded on the surface of their whole function top, but not only on their peak point. For the pyramid shape, if the force is loaded on the peak point, stress concentration will happen and the voltage will be extremely high to 300 V. These results are useless because the absolute pointed shape is inaccessible for industrial production, and a very slender structure is fragile. Therefore, pyramid loading is applied to the four sides vertically, and the force on each side is 5 mN. Similarly, the force loads on the triangular prism side are applied vertically to the two sides as 5 mN. To control variables, the force applied on other structures is all vertically loaded as 5 mN.

### 2.5. Periodical Changing Load Mode

#### 2.5.1. Device Settings

**Figure 3:**
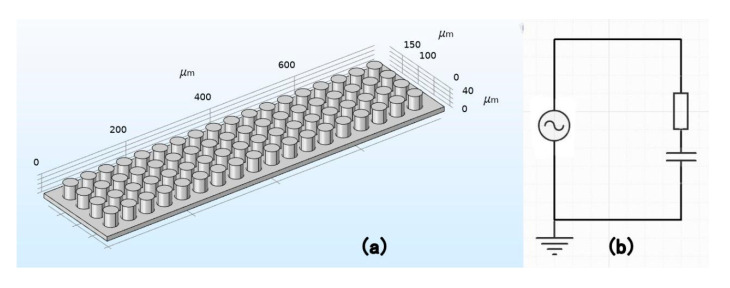
(**a**) Device setting diagram of cylinder modified structure. (**b**) Circuit diagram of the piezoelectric nanogenerator (PENG).

The material is P(VDF-TrFE). The cantilever’s bottom shape is 800 μm in length, 200 μm in width, and 10 μm in height. The function top shape is a cylinder with a radius of 15 μm and a center distance of $2\times \sqrt{2}\times 15$ μm and a height of 30 μm. There are 18 rows and 4 columns of cylinders in the plane, as shown in Figure 3a. The bottom of the structure connects to the ground, and the top cylinder surface connects to the circuit. The circuit is shown in Figure 3b. As shown in Figure 3b, the PENG is viewed as an AC power. The default R is 1000 Ω, and the capacity is 100 nF. Each function top is connected to the circuit and the bottom is also connected to the ground. Therefore, this piezoelectric generator is connected in parallel.

#### 2.5.2. Loading Settings

The force is loaded vertically (only) on the top of the cylinder surface. The first loading function is F×sin(2aπt) in which ‘a’ is the frequency. The periodical changing load makes it easy to observe the response of current and voltage. The ease of controlling frequency also makes it an attractive choice. The second loading function is F×tri(t). tri(t) is a triangular wave, of which the maximum value is 1. Not periodical as it be, the triangular wave is conducive and clear in current-Loading relationship research.

## 3. Result and Discussion

### 3.1. Vertical Load Mode

#### 3.1.1. Experiment Verification

(1) Fabrication of piezoelectric nanogenerators

**Figure 4:**
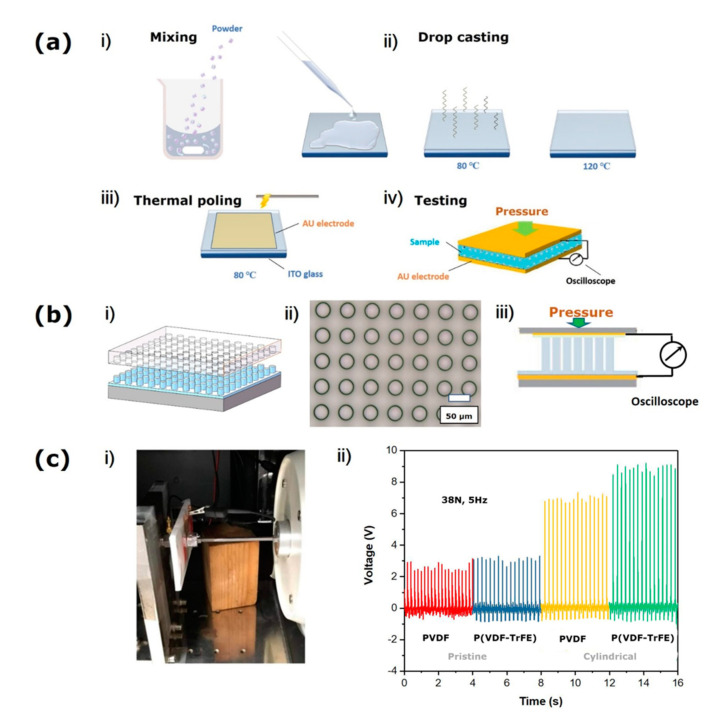
(**a**) (**i**,**ii**) The fabrication of the pristine samples using a low temperature solvent evaporation method, (**iii**) Thermal poling procedure, (**iv**) Schematic diagram of piezoelectric generator. (**b**) (**i**) Schematic diagram of fabrication procedure of the imprinted micropillar aligned P(VDF-TrFE) film on the conductive ITO substrate, (**ii**) The optical microscope images of the imprinted P(VDF-TrFE) micropillars, (**iii**) Schematic diagram of the imprinted micropillar aligned piezoelectric nanogenerator.(**c**) (**i**) The corresponding photograph of the set-up, (**ii**) Voltage output of the pristine structure PVDF and P(VDF-TrFE) and the cylindrical structure PVDF and P(VDF-TrFE) under uniform loading force of 38 N and 5 Hz.

To fabricate the piezoelectric nanogenerator of the pristine (without modified) PVDF structure and P(VDF-TrFE) structure, PVDF(Aldrich) and P(VDF-TrFE) (65 wt.%/35 wt.%, Solvay Solexis US) samples were fabricated using a low temperature solvent evaporation method (as shown in Figure 4a(i,ii)). Firstly, the powders were mixed within DMF (dimethylformamide) solvent with a concentration of 70 wt.% when they were churned at 50 °C for 4 h. Using the drop-cast method, the mixed solution was coated on an ITO(Indium tin oxide) glass, and it was heated to 80 °C to evaporate the solvent. An anneal process was implemented at 120 °C for 2 h. For conducting electricity, the sample was gold coated by sputtering on both sides and later poled on the electric field of 60 MV/m at 80 °C for 2 h. Lastly, the upper and lower sides are connected to copper wires, which can stick to the sample. The electric signal was shown through an oscilloscope when the load was applied (as shown in Figure 4a(iii,iv)).

To fabricate the piezoelectric nanogenerator with micropillar array, P(VDF-TrFE) powder(65 wt.%/35 wt.%) was dissolved in a DMF solvent and spin coated onto an ITO (indium tin oxide)-deposited substrate to produce a film(as shown in Figure 4b). Using the drop-cast method, the mixed solution was coated on an ITO glass, and it was heated to 80 °C to evaporate the solvent. An anneal process was implemented at 120 °C for 2 h. During hot embossing, a PDMS mold arrayed with microcavities was pressed against the P(VDF-TrFE) film under a pressure of 5 MPa at 160 °C for 2 h, resulting in the formation of micropillar array within the micropores of the PDMS mold. Imprinted micropillar aligned P(VDF-TrFE) film on the conductive ITO substrate as the lower electrode was fabricated with a diameter of 30 μm and a height of 50 μm after removing the PDMS mold. Then, a spin-coated thin PDMS layer was put at 5000 rpm for 90 s on the top of the micropillar array. The thin and adhesive dielectric PDMS layer also can stick the upper electrode and maintain the electric stability. The upper electrode was also obtained by sputtering and connected to the copper wires. By the electric circuit, the piezoelectric potential along the micropillar array can drive the free electrons from the low potential domain to the high potential one, so the thin PDMS layer between the top electrode and the micropillars array would not affect the performance. The Polyimide film was sputtered by gold and assembled as the upper electrode and the electric field of 60 MV/m was applied to align the molecular dipoles of piezoelectric P(VDF-TrFE).

(2) Characterization

Optical microscopy was performed on the nanocomposite films using a VHX-600E digital microscope (Keyence Company, Shanghai, China) with a real-time depth composition. An oscilloscope (Tektronix, MDO 3024, Shenzhen, China) was used to measure the voltage outputs. The excitation force was applied by a magnetic shaker (Model JZK-20, Sinocera, Suzhou, China).

(3) Experimental results

The experiment shows the output voltage of the pristine (flat plate) structure PVDF structure and P(VDF-TrFE) structure and the cylindrical structure P(VDF-TrFE), the result is also shown in Figure 4c. The polarization voltage of both materials is 60 MV/m, the force applied on the surface is 38 N, and the frequency is 5 Hz. The voltage output of the pristine PVDF and P(VDF-TrFE) are 2.6 V, 3.1 V, respectively. After micro modifying, the voltage output property increases obviously and the value reaches 8.8 V. The ferroelectric all-trans planar zigzag molecular chains were configurated, and the available switching dipoles appeared. When the force was applied, the molecular dipoles of samples aligned, and the dipole density changed. The free-charge carriers moved from the negative potential to the positive potential region to balance the electric potential, and a positive voltage peak was produced. The superior performance of the sample based on micropillars can be attributed to the enhanced mechanical flexibility of the micropillar array under compression [44].

#### 3.1.2. Simulation Result

**Figure 5:**
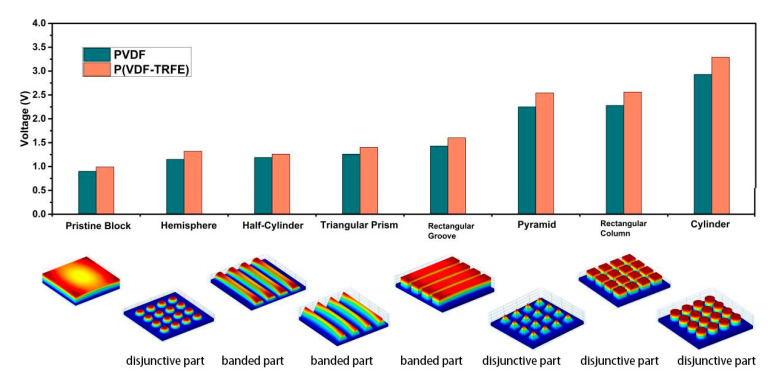
Voltage comparison of eight structures. The small diagrams below are voltage nephogram of eight designs. The left coordinate axis denotes the voltage output.

Figure 5 shows the results of the simulation of eight structures. As shown in the diagram, the cylinder has the largest voltage output. The flat pristine structure has the lowest output. P(VDF-TrFE) produces a higher voltage than PVDF does under the same loading. Bright color denotes higher voltage, and darker color represents a lower voltage. Because the bottom structure is connected to the ground, the bottom voltage is normally zero.

The simulation results show thatthe pyramid voltage is 2.54 V (P(VDF-TrFE)) and 2.25 V (PVDF), and that the cylinder voltage is 3.3 V (P(VDF-TrFE)) and 3 V (PVDF). This is similar to the experiment work. In Lee’s experiment [46], the output is 3.8 V (P(VDF-TrFE)). The P(VDF-TrFE) function shape of Lee’s experiment work consists of the pyramid and triangular prism, which produces 4.4 V and 3.6 V respectively under 1.65 kgf (about 16 N), which also shows that the pyramid is better than triangular prism shape. The results from Chen et al. [39] show that the pristine voltage of 1.99 V and cylinder voltage is about six times to the pristine voltage. The height of the cylinder is 55 μm and the base thickness is 5 μm, in which the height of the pristine structure is 60 μm. The loading force is 5 mN. The diameter of the cylinder is 22 μm. His voltage output is slightly larger than the outcome in this paper, but considering the height of their structure is 60 μm, where normally a higher structure produces higher peak voltage. The voltage output after modification is three times that of the pristine structure, which proves the surface shape-modification is beneficial and indispensable.

#### 3.1.3. Simulation Result Discussion

From the results above, some discussions can be achieved.

**Figure 6:**
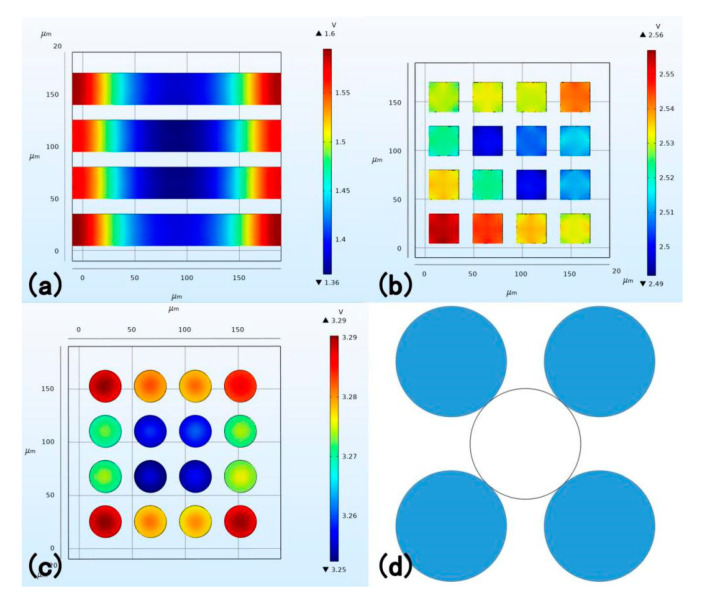
Top view of the voltage. (**a**) Top view of the rectangular groove (**b**) Top view of the rectangular column (**c**) Top view of the cylinder. (**d**) Top view of the cylinder pillar allocation.

(1) The smaller surface area can achieve a higher voltage output, the reason is that the loading is a fixed force, thus the smaller the surface area, the larger the pressure and thus the larger voltage output. But reducing the support area might also decrease the reliability and stability.

(2) The disjunctive part produces a larger voltage than the banded part. On the one hand, this is because of the smaller base area, which produces larger pressure. On the other hand, the disjunctive part gathers local extreme value better, comparing to the banded part which only acquires local peak voltage at the edge of the overall structure. (As shown in Figure 6, in which bright color denotes high voltage and darker color denotes low voltage). The only exception, the semi-sphere structure, whose voltage is lower than banded groups’ as a disjunctive one, resulting largely to its height. Figure 6a–c are rectangular groove shape, rectangular column shape and cylinder shape, respectively.

(3) The disjunctive function part is conducive for uniformed voltage distribution, which manifestly has a widespread application in various aspects. The local peak voltage appears mostly at the pointed edge, and thus the banded parts encounter a large decrease and then a large increase. Such oscillation of voltage sets higher criteria for electric facilities and sometimes destroys them. Here, the ratio of the difference value of the peak voltage and the least value to the least value within a single plane is defined as a voltage fluctuation rate (VFR). VFR is a variable for measuring the uniform distribution characteristics of the PENG, in which a lower VFR represents a uniform voltage distribution. Suppose that the following process utilizes the top voltage mainly, the VFR of the rectangular column is 2.81%, for the cylinder is 1.23% and for the rectangular groove is 17.35%. (As shown in Figure 6b,c)

(3) $$VFR=\frac{V_{\max}-V_{\min}}{V_{\min}}\times 100\%$$

(4) For the disjunctive part, a better geometric symmetry is favorable. Here, the VFR in a single function block is called SBVFR (single block voltage fluctuation rate) (in one certain plane as well). SBVFR is a variable measuring voltage distribution uniformity in one function top. A lower SBVFR, apparently, represents a uniform distribution. The SBVFR of the cylinder is 0.12%, for the rectangular column is 0.37%. Therefore, a better geometrical symmetry is helpful for voltage stability. Meanwhile, one exclusive nature of the cylinder is that the peak voltage appears at the center. Part of the reason for the cylinder’s high voltage is attributed to its symmetry, which concentrates the charge in a restrained area, compared to other structures such as rectangular, which assembles charge in four vertices.

#### 3.1.4. Parameters Improvement Based on Cylinder Structure

Considering its outstanding voltage output, the cylinder structure has therefore been researched. Four parameters are discussed through COMSOL 5.4. The pressure applied on the top is the same. The material is P(VDF-TrFE).

(1) The relationship between radius and the voltage output

**Figure 7:**
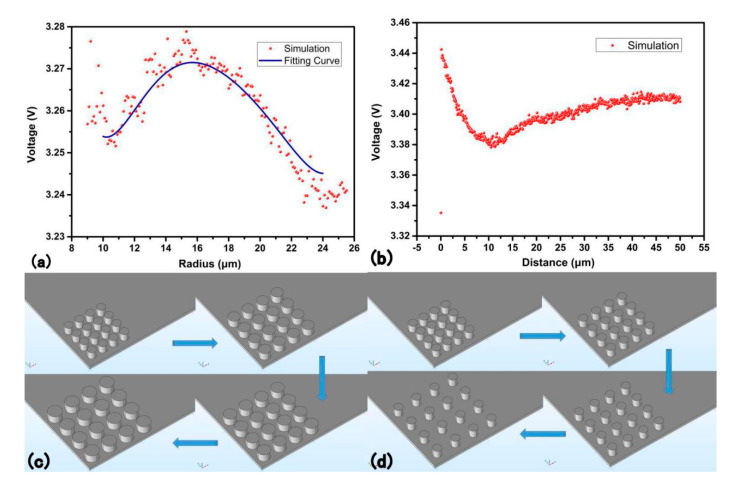
(**a**) Relationship between the radius and the voltage. (**b**) Relationship between the distance and the voltage. (**c**) The concept diagram of the radius changing. The circle pillar follows five-circle tangent allocation. (**d**) The concept diagram of the distance changing. The diameter keeps constant as 30 μm here and the distance sweeps from 0 to 50 μm.

Figure 7a is the diagram of the radius and the voltage output, and Figure 7c is the concept diagram of the radius changing in which the radius follows the five-circle-tangent allocation rule. Here, the ratio of function area to the total support base area is called unit effective area (UEA), which is positively correlated with the radius. When the UEA is low, the voltage production will be difficult and when it is large, the interference transmitted by the support base will increase. Therefore, a suitable radius should be considered. It is reasonable that if the support base area is infinite, the voltage will not be influenced by the radius. However, that is impossible in reality. Figure 7a shows that the best radius should be considered in different cases.

(2) The relationship between the distance of the cylinder and the voltage output

To control variables, the loading on the function top is set as pressure because the radius is changing and thus the area is changing. The number and allocation of all cylinders are all set as 4 × 4 (rows time columns). The radius of the cylinder is 15 μm.

Figure 7b,d are both the relationship between the distance (between cyinders) and the voltage output, in which the radius of the cylinders is fixed. As shown in Figure 7b, with the increase of the distance, the voltage undergoes a decrease and then undergoes an increase. When the distance (circle center distance minus diameter) between the cylinders is 0, which is similar to pristine structure, the voltage is manifestly lower than the normal modified cylinder structure, matching well with the prediction. The voltage value at 0 distance is still larger than the pristine structure (0.99 V) attributes to the interval caused by the cylinder’s shape itself. It can be inferred that an interaction transmitted by the supporting base exists, which can be seen as two adversely functioning forces. As the distance increases, the force facilitating the voltage increases and the force to cause interfere decreases. Before the local extreme value, the facilitating force effect is dim but the situation reverses after the local extreme value. The function works similar to the interatomic force. Here, the local extreme value appears at approximately 10 μm.

(3) The relationship between height (depth-to-width ratio) and voltage output

**Figure 8:**
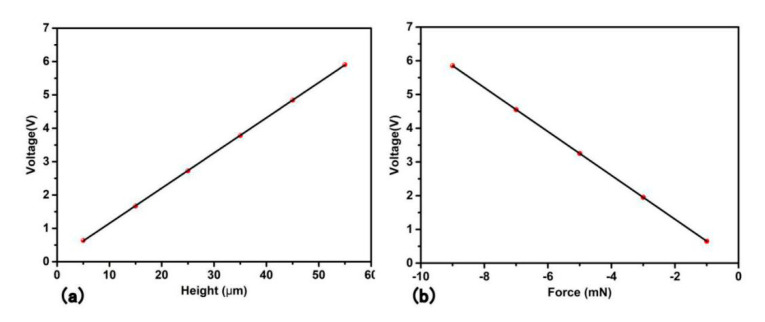
(**a**)The relationship between height and voltage. (**b**) The relationship between force and voltage. The minus force denotes the direction of the force goes in the normal direction to the inner part of the structure.

Figure 8a shows the relationship between height and voltage output. The radius and distance between cylinders are fixed, therefore, the change of the height is equal to the change of depth-to-width ratio. With the height increases, the voltage output increases at the same time. The linear relationship between height and voltage is quite clear. It is obvious that thin and tall pillar is beneficial for a higher voltage.

(4) The relationship between loading force and voltage output

Figure 8b is the relationship between the force and the voltage output. When the force increases, the voltage output increases linearly. It is easy to prove that the extension line goes across the origin. As shown in Figure 8b, the force loading is linear to voltage output and fits well to the fitting curve. The force is minus because the direction of the force is contradicted to the positive direction of the surface. It is reasonable that the extension line will go through the origin because without force loading, the voltage cannot be produced. Chen’s experiment also finds a linear relationship. With the bottom fixed, the cylinder modified PENG structure is loaded vertically through a trapezoid bump [47]. Lee’s experiment also shows a linear relationship between pushing force and output voltage. The shape also consists of the support base and function top. The linear relation is the clearest in flat shape and the pyramid and the triangular prism intercept of y axis is actually higher than the origin, but still follows the linear relationship [41].

### 3.2. Periodical Changing Load

Changing load is common in PENG design. Different from vertical load mode, changing mode could produce current, because the load does work to the structure. Additionally, the periodicity of the load will make the research process clearer.

As the amplitude of the load is fixed, the frequency of the load is one of the key considerations here. The frequency sweep list is divided into high frequency group and low-frequency group. The voltage output of the cantilever PENG has some relationships with eigenfrequency [43], so the frequency sweep list is based on the eigenfrequency. The first 5 mode resonate frequencies are 4398 Hz, 27,438 Hz, 39,325 Hz, 66,926 Hz, 76,376 Hz (calculated through COMSOL 5.4) (Table 1 High-Frequency Group). According to the research of Alexandre et al., the resonant frequency decreases with the length, the high frequency of the micro-structure in this paper is reasonable. A low-frequency group is added considering the practical usage: 10 Hz, 50 Hz, 80 Hz, 100 Hz and 300 Hz. The eigenfrequency is calculated by COMSOL 5.4. The low-frequency is 10 Hz, 50 Hz, 80 Hz, 100 Hz, 300 Hz, 500 Hz (Table 1 Low-Frequency Group). The loading function is F×sin(2aπt), in which ‘a’ is the frequency.

#### 3.2.1. Simulation Result

**Figure 9:**
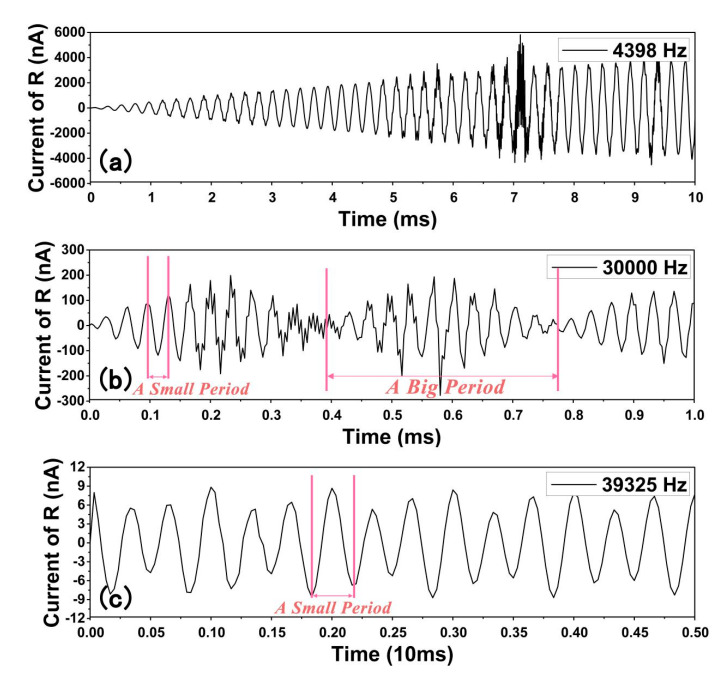
(**a**) ‘Tip shape’ relationship. Current response of the cylinder modified structure under 4398 Hz. (**b**) ‘Fusiform shape’ relationship. Current response diagram of ‘Big period’ and peak value of 30,000 Hz with cylinder modified-structure. (**c**) Current response diagram of pristine structure at 39,325 Hz. (Complete diagrams are all listed in the support information) (The x-axis of the three diagrams are all ‘time(s)’). (Current of R shows the current of the resistance).

Figure 9 shows the current response of time of cylinder modified and pristine structure. Figure 9a is the tip shape mode of the cylinder modified current response, which enters the stable stage slowly and then experiences a stable stage like pristine shape. ‘Tip shape’ means that when viewed horizontally, the shape of the curve is similar to that of a pointed point. Another pattern of cylinder modified structure is Figure 9b, ‘fusiform shape’, of which the middle is wider and the ends are thinner when it is viewed horizontally. This mode’s peak value of the current increases with frequency (as shown in Figure 9a). The maximum current value also experiences a decrease and increases with frequency as shown in Figure 9b. ‘Small period’ is the normal period, and the ‘big period’ is an aggregation of the periodic rise and fall of the peak current, which consists of lots of small periods, which are shown in Figure 9b,c respectively. The value of each small period in Figure 9b is not completely coincided with the frequency, which means if the trembling frequency increases, the current-response frequency will not increase at the same rate. This is completely different from the pristine structure, which strictly coincides with the trembling frequency.

**Figure 10:**
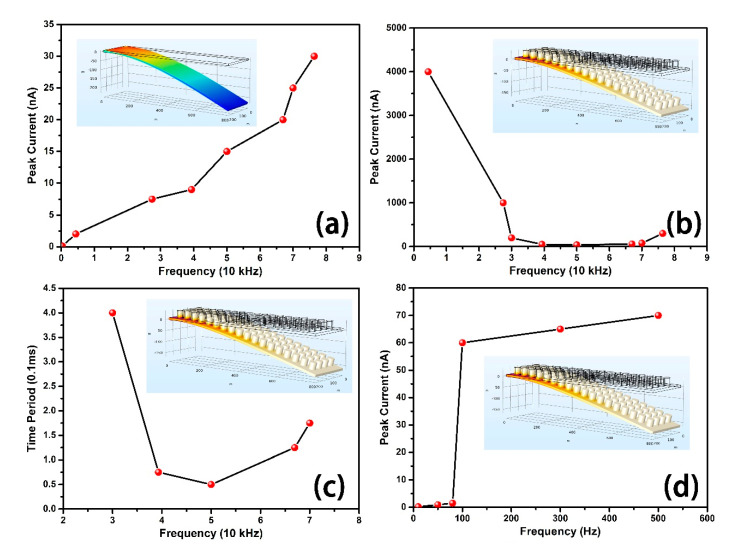
(**a**) Peak current diagram of pristine structure (**b**) Peak current of cylinder modified structure (**c**) Big period value of the cylinder modified structure. All the red points above are simulation results and black lines are the fitting curve. (**d**) Peak current of low frequency.

Additionally, the pristine structure’s current is all like Figure 9c, of which peak current value is quite stable. The increase of the peak current value and the decrease of the current value is redistribution of the energy with time. Although such current instability might be harmful to electric devices, but for some devices which needs high current or voltage output to convey signal, this property is useful. The tip shape mode such as 4398 Hz, which produces a stable peak value after long preparation time, can be more valuable for electric devices which need high current and stable current value. The fusiform shape mode such as 30,000 Hz, which produces a large peak current periodically, can be more useful for sensor design. The energy density under 4398 Hz can be calculated as 1 × 10^−2^ W/m^2^, some comparisons with previous studies are listed below in Table 2. Besides, the response frequency of the modified structure is quite similar to the input load frequency. The response frequency of the modified structure can be calculated using Figure 10c, where the response frequency is the reciprocal of the time period. The power of both the modified and the unmodified structure can be calculated using I^2^R formula.

#### 3.2.2. Simulation Result Discussion

Figure 10a shows the relationship between frequency and the peak current value of the pristine structure. The circuit consists of a generator (PENG), a resistor(R), and a capacitor(C). The y value is “the current of R”, which means the current is measured at R. The peak current increases with the frequency almost linearly. Figure 10b shows the relationship between peak current value and the frequency of the cylinder modified structure, of which the peak current value decreases with frequency before 5 × 10^4^ Hz and increases after 5 × 10^4^ Hz. Figure 10c also shows a ‘U’ shape of the relationship between the time period and the frequency of the cylinder modified structure, of which the minimum point appears around 5 × 10^4^ Hz, similar to the minimum point of the peak current mentioned before. Figure 10d shows the peak current output of the improved structure under low-frequency. The current under frequency lower than 100 Hz is relatively low and it increases quickly at 200~300 Hz.

The performance improvement comes from the micro-pillar structure itself. There have been some studies implementing shape modification on the cantilever, and one of the improvement methods is to add mass block on the tip away from the fixed end on the cantilever. The difference is that the vibrating stages of the cylinder pillar on the surface are complicated. This partly interferes with the response of the peak current to the eigenfrequency and hides the existence of the resonance current. The existence of the micro-pillar also delays the coming of the peak current, because it takes time for all the pillars to vibrate accordingly and produce periodical current, which shapes the ‘big period’ and the ‘small period’. At the same time, the pristine shape does not need such ‘preparation time’. The wide range peak value fluctuation feature and the slow peak value entering feature limits cylinder modified structure application on some devices which need stable current. However, considering most of the PENGs store energy on a capacity through a controllable chipset, this limitation is acceptable. It seems that the enhancement of the current of the cylinder modified structure is surprisingly high, but the truth is, it reallocates the distribution of the energy, in which the small peak of the cylinder modified structure could reach 0 and is much smaller than the pristine structure.

The small period does not respond with the frequency as the pristine structure does, which increases with the frequency precisely. Additionally, the pristine structure produces a fixed peak current at a specific frequency. The results show that the eigenfrequency is of little influence to the current output. The enhancement of the current is significant. For 30 kHz, the peak current of the improved structure is 20 times larger than the pristine structure. A frequency that is lower than 4398 Hz or higher than 76,376 Hz is valuable for a long time working because of the large current output.

Futhermore, the structure is tested at low-frequency. The test results show that the current of the cylinder modified structure increases greatly compared with the original structure, such as nearly 400 times at 300 Hz (original design: 0.15 nA, modified design: 65 nA). In the case of low-frequency, there is no “big period” phenomenon as in the case of high frequency, because the vibration frequency is too low to make the surface microcolumn vibrate adequately. The above explanation is further supported by the observation that the peak current remains unchanged below 100 Hz.

#### 3.2.3. Electrical Loading Circuit Properties

Four electrical loading circuit properties of the structure are simulated and tested by COMSOL 5.4.

(1) The suitable resistance

**Figure 11:**
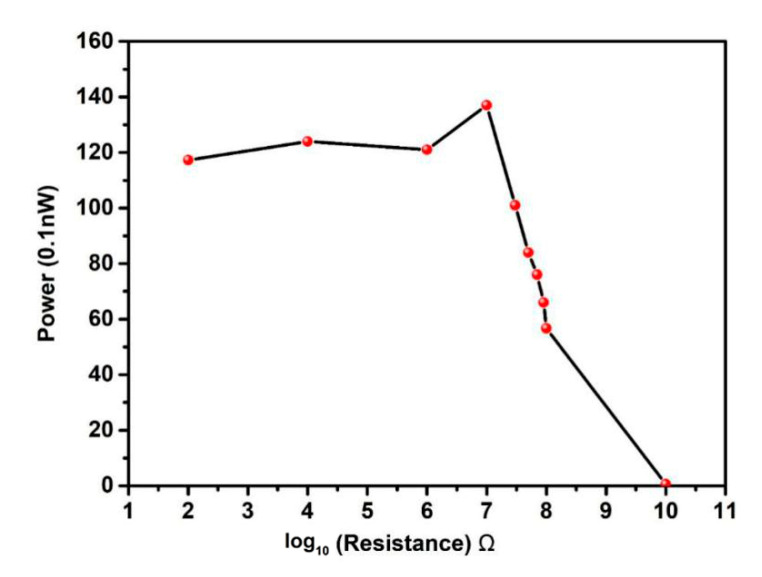
The relationship between resistance and power output. The optimized resistance is found to be around 10^7^ Ω. It is so large because of the simulated structure’s scale is on the micron scale.

Figure 11 shows the relationship between resistance and power output. The data in Figure 11 are extracted from current-time response graph. The current increases with resistance at first and decreases with the resistance then. The peak value appears at 10^7^ Ω. Considering that the x-axis data has been processed by log_10_, the power value before 10^7^ Ω does not increases as slowly as the resistance value as shown in the Figure 11, and the power declines after 10^7^ Ω is not that fast, but it is clear that at around the resistance value of 10^10^ Ω, the power is almost zero. The resistance changing trend is similar to Chen’s paper, which implements the experiment and gets a suitable value as well. Lee’s paper shows that in an experiment, the suitable resistance is achieved around 10^7^ Ω. The model of their paper consists of the support base and pyramid function top shape [46].

(2) Synchronization of current and voltage

**Figure 12:**
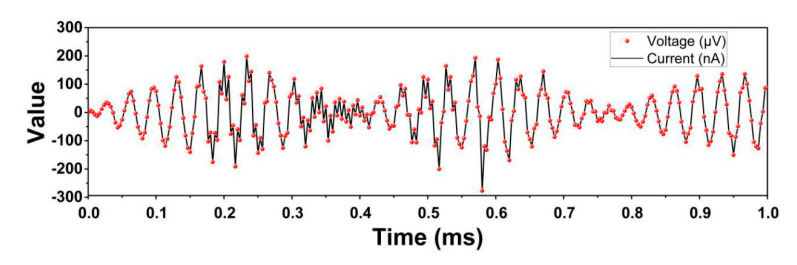
Current–voltage response diagram under sine wave (of cylinder modified structure).

The current and the voltage response to time are found to be at the same frequency, and the value of voltage is 1000 times as great as the current, which coincides with the ohm law, as shown in Figure 12. The current is positively related to loading, which is quite hard to implement in real tests because the time interval of the real test is often within a millisecond. The current is in direct proportion to the loading force if a small time-interval is added to the current.

(3) The relationship between loading speed and current

**Figure 13:**
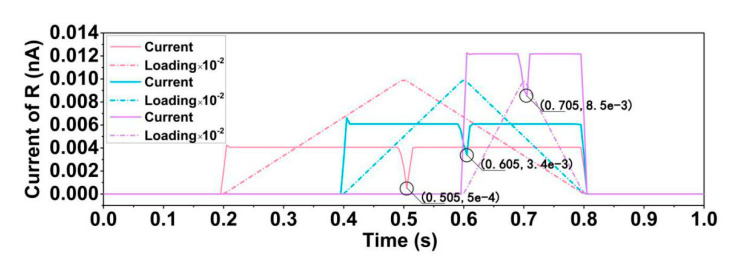
Current response diagram under triangular wave loading with three different loading slopes. Each color represents a different loading type. The black circle shows where the peak value arrives. The triangular waves reach the peak at 0.5 s, 0.6 s, and 0.7 s, respectively. The peak values of the loading are the same. (The current is the absolute value here) (Current of R shows the current of the resistance).

**Figure 14:**
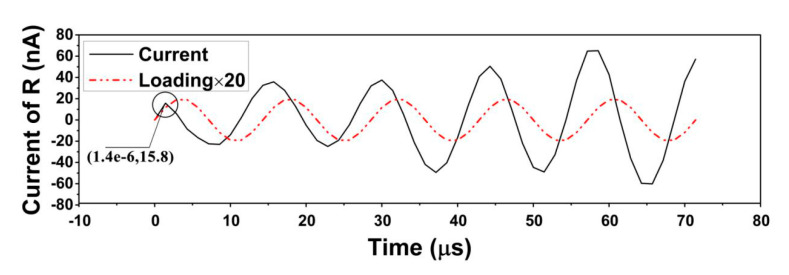
Loading-current response diagram (of 70,000 Hz). The loading is multiplied by 20 to make it easier to observe. (Current of R shows the current of the resistance).

Figure 13 shows the relationship between the current and loading of the cylinder modified structure under a triangular wave loading. When the derivative of the loading increases, the current increases accordingly. Additionally, Figure 14 shows the current responses to the loading of the cylinder modified structure under the sine loading. The loading here follows sine rule, and it turns out that the current follows a cosine rule (if added small time interval). Both tests show a positive relationship between the current and the loading. The energy density could be calculated through the power output.

(4) Time delay property

The time interregnum of peak loading to local peak current is correlated with loading rule. As shown in Figure 13, the y-axis denotes the absolute value of the current and the normal value of the triangular wave for the sake of easy observation and comparison of the local peak current value. When the loading increases, the current is positive and when the loading decreases, the current is negative. The peak of the loading arrives at 0.5, 0.6 and 0.7 s, the local peak value of the current arrives at 0.505, 0.605, 0.705 s. Figure 14 shows a time delay of 1.4 × 10^−6^ s.

## 4. Conclusions

In this work, the piezoelectric properties of micro-modified structure of PVDF and its copolymer P(VDF-TrFE) is compared to the pristine structure under vertical load and the periodical changing load using finite element method. The influence of surface-modified structure characteristics on electrical properties of energy harvester is systematically analyzed based on finite element method.

At the vertical load mode, eight structures are designed and compared, the voltage output sequence under the same loading force are: pristine block < hemisphere < half-cylinder < triangular prism < rectangular groove < pyramid < rectangular column < cylinder. The voltage output of cylinder shape is 3 times that of the pristine shape. For the function top, a disjunctive pillar is better than a banded structure. A highly symmetrical function top shape is preferred. Relevant experiment has been done to verify the simulation. The structure improvement discussion is done under the cylinder pillar function top shape. The relationship between radius, height, force and distance to the voltage output of the cylinder allocation are discussed. The voltage output decreases with distance (center distance minus diameter) before 10 μm and increases after that. The voltage output increases with the radius of the cylinder and then decreases. The best radius is 16 μm for this specific structure. The height and the force loading are positively related to the voltage output.

At the periodical changing load mode, the current output of the cylinder modified structure represents some distinctive features. There are two response modes: the tip shape mode and the fusiform shape mode. For the fusiform shape mode, the peak current and the big period decreases with the frequency and increases then. For the tip shape mode, the current peak increases slowly and then keep constant. The ‘big period’ of tip shape mode could be seen as infinite and thus when considering the two modes, the length of the big period decreases with the increasing of the frequency and then increases, forming a ‘U’ shape diagram. Because the peak current of the tip shape is larger than the fusiform, the diagram is also ‘U’ shape. The cylinder modified structure also shows an apparent current output improvement under low-frequency, for 80 Hz, the improved structure is 50 times than pristine one.

The eigenfrequency is of little influence to the response outcome. The cylinder modified structure is of great importance to the current output, and for 30 kHz, the enhancement is 20 times. For pristine structure, the peak current is fixed at a specific frequency and the response period is precisely correlated with the increasing of the frequency.

Four electrical loading circle properties are discussed. The suitable resistance of these devices is around 10^7^ Ω, and the current synchronizes to the voltage. The current is positively correlated with the loading and a time delay of current response to loading has been found.

## Figures and Tables

**Table 1:** Vibration frequency list.

	1	2	3	4	5	6	7	8
High-Frequency Group (Hz)	4398	27,438	30,000	39,325	50,000	66,926	70,000	76,376
Low-Frequency Group (Hz)	10	50	80	100	300	500		

**Table 2:** Comparison of energy output between this research and previous research.

	Piezoelectric Materials	Key Parameters	Resonant Frequency	Output Power (per Area)	Output Power (per Voltage)
This work	P(VDF-TrFE)	Length = 200 μm; Width = 800 μm; Thickness = 10 μm;	4398 Hz	1 × 10^−^^2^ W/m^2^	1 × 10^3^ W/m^3^
Work 1 [48]	PZT	Length = 3 mm; Width = 5 mm;	618 Hz	6.267 × 10^−^^3^ W/m^2^	
Work 2 [49]	PZT	Length = 13.5 mm; Width = 9 mm; Thickness = 192 μm;	229 Hz		0.0114 × 10^3^ W/m^3^
Work 3 [50]	PZT	Length = 3000 μm; Width = 1500 μm; Thickness = 22 μm;	575 Hz		0.4758 × 10^3^ W/m^3^
Work 4 [51]	PZT	Length = 1600 μm; Width = 400 μm; Thickness = 30 μm;			0.1 × 10^3^ W/m^3^
Work 5 [35]	P(VDF-TrFE)	Length = 1000 μm; Width = 300 μm; Thickness = 2.5 μm;	477.03 Hz		0.24992 × 10^3^ W/m^3^
